# Systemic Inflammatory Markers Are Closely Associated with Atherogenic Lipoprotein Subfractions in Patients Undergoing Coronary Angiography

**DOI:** 10.1155/2015/235742

**Published:** 2015-11-25

**Authors:** Yan Zhang, Sha Li, Rui-Xia Xu, Cheng-Gang Zhu, Yuan-Lin Guo, Na-Qiong Wu, Jing Sun, Jian-Jun Li

**Affiliations:** Division of Dyslipidemia, State Key Laboratory of Cardiovascular Disease, Fu Wai Hospital, National Center for Cardiovascular Diseases, Peking Union Medical College, Chinese Academy of Medical Sciences, BeiLiShi Road 167, Beijing 100037, China

## Abstract

*Objective*. To investigate the relationship between inflammatory markers and atherogenic lipoprotein subfractions.* Methods*. We studied 520 eligible subjects who were not receiving any lipid-lowering therapy. The inflammatory markers including white blood cell (WBC) count, high-sensitivity C-reactive protein (hs-CRP), fibrinogen, erythrocyte sedimentation rate (ESR), and D-dimer were measured. A multimarker inflammatory index was developed. Low-density lipoprotein (LDL) and high-density lipoprotein (HDL) separation processes were performed using Lipoprint System.* Results*. In age- and sex-adjusted analysis, several inflammatory markers (WBC count, hs-CRP, fibrinogen, and ESR) were positively related to circulating non-HDL cholesterol and remnant cholesterol (*p* < 0.05, all). Among lipoprotein subfractions, we observed a positive association of inflammatory markers with very low-density lipoprotein cholesterol, small LDL cholesterol, and LDL score (*p* < 0.05, all). Meanwhile, a negative association was detected between inflammatory markers and mean LDL particle size (*p* < 0.05) or large HDL cholesterol (*p* < 0.05). Moreover, we found that the relationships between multimarker index quartiles and small LDL cholesterol, LDL score, and mean LDL particle size were slightly stronger in patients with CAD.* Conclusions*. Systemic inflammatory markers are positively correlated with small LDL cholesterol and LDL score while being negatively linked with mean LDL particle size and large HDL cholesterol, highlighting the potential contribution to increased cardiovascular risk.

## 1. Introduction

Coronary artery disease (CAD) represents an important public health burden. Aside from the traditional risk factors, compelling evidence has now accumulated in support of inflammation as an important risk factor [1]. It has a pivotal role in all stages of atherosclerosis from endothelial dysfunction and plaque formation to plaque disruption and thrombosis [2]. Several inflammatory markers such as C-reactive protein (CRP) and fibrinogen have acquired prognostic significance [3, 4].

Although dyslipidemia has been recognized as the major cardiovascular risk factor and lowering of low-density lipoprotein (LDL) cholesterol has convincingly been shown to reduce cardiovascular events, there is still considerable remaining risk [5, 6]. Indeed, LDL particles are a heterogeneous collection of particles which vary in potential pathologic properties such as size, density, and lipid composition [7]. Moreover, high-density lipoprotein (HDL) particles are much more heterogeneous in their size and composition than LDL. This may at least partly explain why attempts to reduce cardiovascular events by pharmacologically increasing HDL plasma levels have failed [8, 9]. Hence, lipoprotein subfractions have emerged as a novel approach to assess the atherogenicity of lipoproteins.

There is a large body of evidence linking inflammatory status and dyslipidemia [10, 11]. Notably, it has been well established that inflammation is also able to affect lipoprotein metabolism [12]. Moreover, our previous data demonstrated positive associations between inflammatory markers and important cholesterol regulator, proprotein convertase subtilisin/kexin type 9 (PCSK9) [13, 14], which has also been found to be closely related to atherogenic lipoprotein subfractions [15, 16], However, whether there are certain relationships between inflammatory markers and lipoprotein subfractions has not been reported yet. To address this question, we studied five kinds of systemic inflammatory markers (white blood cell (WBC) count and its subsets, high-sensitivity CRP (hs-CRP), fibrinogen, erythrocyte sedimentation rate (ESR), and D-dimer) in a group of subjects scheduled for coronary angiography who were not receiving any lipid-lowering therapy. Specifically, we hypothesized a presence of heterogeneity in the relationship of systemic inflammatory markers with atherogenic lipoprotein subfractions, which would aid our understanding of their interplay in the pathogenesis of atherosclerotic disease.

## 2. Materials and Methods

### 2.1. Study Design and Population

The study complied with the Declaration of Helsinki and was approved by the hospital's ethical review board (Fu Wai Hospital, National Center for Cardiovascular Diseases, Beijing, China). Each participant provided written, informed consent before enrollment.

**Figure 1:**
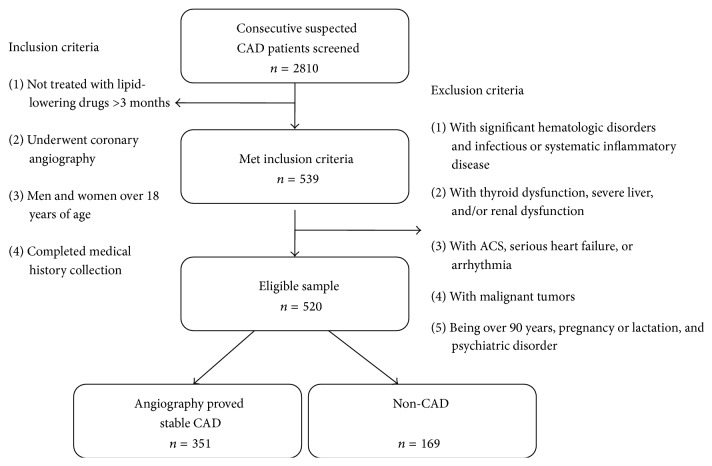
The flowchart of the current study.

In a group of subjects scheduled for coronary angiography because of angina-like chest pain and/or positive treadmill exercise test or clinically suspected CAD in our department, we selected 520 consecutive individuals who were not treated with lipid-lowering drugs (351 CAD and 169 non-CAD). Inclusion criteria were as follows: (1) having no treatment history of statins and/or other lipid-lowering drugs at least 3 months before entering the study; (2) having detailed clinical, laboratory data and well documented traditional cardiovascular risk factors; (3) having undergone coronary angiography. Exclusion criteria were subjects over 90 years, pregnancy or lactation, psychiatric disorder, the existence of any infectious or systematic inflammatory disease, acute coronary syndrome, serious heart failure or arrhythmia, significant hematologic disorders, thyroid dysfunction, severe liver dysfunction, and/or renal insufficiency and malignant tumors. The flowchart of the current study was shown in Figure 1.

Hypertension was defined as repeated blood pressure measurements ≥140/90 mmHg (at least two times in different environments) or currently taking antihypertensive drugs. Diabetes mellitus (DM) was defined as a fasting serum glucose level ≥126 mg/dL in multiple determinations and/or the current use of medication for diabetes. Dyslipidemia was defined by medical history or the use of lipid-modulating medications in order to reduce lipids or fasting total cholesterol (TC) ≥200 mg/dL or triglyceride (TG) ≥150 mg/dL.

### 2.2. Biochemical and Clinical Analyses

Fasting blood samples were collected in precooled EDTA tubes at baseline from each patient. After centrifugation at 3000 rpm for 15 min at 4°C, all plasma aliquots were stored in our laboratory at −80°C and were not thawed until use.

The concentrations of lipid profiles were determined by automatic biochemistry analyzer (Hitachi 7150, Tokyo, Japan). In detail, the LDL cholesterol concentration was analyzed by selective solubilization method (low-density lipid cholesterol test kit, Kyowa Medex, Tokyo). HDL cholesterol concentration was determined by a homogeneous method (Determiner L HDL, Kyowa Medex, Tokyo). Non-HDL cholesterol was calculated as TC minus HDL cholesterol. Remnant cholesterol was calculated as TC-HDL-C-LDL-C as previously reported [17]. The WBC count, neutrophil, lymphocyte, and monocyte differentials were determined using an automated blood cell counter (Beckman Coulter Ireland Inc., Mervue, Galway, Ireland). The plasma hs-CRP levels were determined using immunoturbidimetry (Beckman Assay 360, Bera, CA, USA). The fibrinogen levels were quantitatively measured by the method of Clauss and a Stago autoanalyzer with STA Fibrinogen kit (Diagnostica Stago, Taverny, France). The Westergren method was used for the measurement of ESR. Plasma D-dimer level was measured by Stago evolution (France).

### 2.3. LDL Subfraction Analysis

The cholesterol contents of LDL subfractions were determined electrophoretically by the use of high-resolution 3% polyacrylamide gel tubes and the Lipoprint LDL System (Quantimetrix Corporation, Redondo Beach, CA, USA) according to the manufacturer's instructions as previously described [18]. Seven LDL subfractions were obtained. Subfraction 1 represented large LDL particles, subfraction 2 indicated intermediate LDL particles, and subfractions 3–7 were defined as small LDL particles. The cholesterol mass (mg/dL) of each lipoprotein subfraction, the mean LDL particle size (Å), and the proportion (%) of the cholesterol mass of each lipoprotein subfraction over the TC mass were determined by this assay. The LDL score was calculated as the proportion of small LDL particles to the whole LDL area in our sample [19].

### 2.4. HDL Subfraction Analysis

Similar to LDL subfraction analysis, the cholesterol contents of HDL subfractions were also determined electrophoretically by the use of high-resolution 3% polyacrylamide gel tubes and the Lipoprint HDL System (Quantimetrix Corporation, Redondo Beach, CA, USA) as in our previously described work [20]. The relative area for each HDL subfraction was determined and multiplied by HDL cholesterol concentration of the sample to yield the amount of cholesterol for each band in mg/dL. Using this assay, HDL was divided into 10 subfractions. Subfractions 1–3 represented large HDL particles, subfractions 4–7 indicated intermediate HDL particles, and subfractions 8–10 meant small HDL particles.

### 2.5. Statistical Analysis

The data were expressed as the mean ± SD for the continuous variables and the number (percentage) for the categorical variables. Student's *t*-test or Mann-Whitney *U* test was used for the comparisons between continuous variables, and the chi-squared test was applied for the categorical variables between CAD and non-CAD group. Correlations between multiple inflammatory markers and lipoprotein subfractions were examined by partial correlation analysis with adjustments for age and sex. The chronic inflammatory activity can be assessed by a number of correlated parameters, such as WBC count, hs-CRP, fibrinogen, and ESR. Hence, we employed a principle component analysis to extract from the individual markers of inflammation (WBC count, hs-CRP, fibrinogen, and ESR) a single weighted multimarker inflammatory index. In the current study, only the first principle component was observed and no additional significant principal components were identified. Accordingly, we developed the overall multimarker inflammatory index by weighting the respective coefficients of each of the four inflammatory markers that contributed to the primary underlying factor (inflammation) as previously reported [21]. The general linear model was used for the comparison of lipoprotein subfractions according to multimarker index quartiles. The categorical variables were compared using the chi-squared test. A *p* value of less than 0.05 was considered statistically significant. Statistical studies were carried out with the SPSS program (version 19.0, SPSS, Chicago, Illinois, USA).

## 3. Results

### 3.1. Baseline Characteristics

A total of 520 individuals were enrolled in the present study. The mean age of the study cohort was 56.6 ± 10.0 years and 335 (64.4%) study participants were male. Among them, 351 (67.6%) had significant angiographically documented CAD as having >50% diameter stenosis in ≥1 major epicardial coronary artery. The main demographic and clinical characteristics of the study subjects are listed in Table 1. As a result, we observed that the CAD group has relatively higher small LDL cholesterol levels (9.0 ± 9.8 versus 7.9 ± 9.3 mg/dL, *p* = 0.092) and LDL score (0.14 ± 0.13 versus 0.12 ± 0.13%, *p* = 0.067) but smaller mean LDL particle size (266.4 ± 5.9 versus 267.2 ± 6.0 Å, *p* = 0.079), although the difference does not reach statistical significance in the current analysis. Meanwhile, the CAD group has dramatically lower large HDL cholesterol (13.5 ± 7.2 versus 15.1 ± 7.7 mg/dL, *p* = 0.027). In addition, several inflammatory markers are increased in patients with CAD, such as WBC count (6.2 ± 1.8 versus 5.9 ± 1.4 (×10^9^/L), *p* = 0.076), neutrophil count (3.8 ± 1.4 versus 3.5 ± 1.1 (×10^9^/L), *p* = 0.019), hs-CRP (2.8 ± 3.1 versus 2.2 ± 2.8 mg/L, *p* < 0.001), and fibrinogen (3.2 ± 0.8 versus 3.0 ± 0.6 g/L, *p* = 0.001).

### 3.2. Correlations of Multiple Inflammatory Markers to Lipoprotein Subfractions

We next determined the strength of the relationship of multiple inflammatory markers with atherogenic lipoprotein subfractions. As shown in Table 2, after adjusting for age and sex, positive associations were observed between inflammatory markers and very low-density lipoprotein (VLDL) as well as intermediate-density lipoprotein (IDL). Among LDL subfractions, small LDL cholesterol was closely and positively related to WBC count (*p* < 0.01), neutrophil count (*p* < 0.05), lymphocyte count (*p* < 0.01), hs-CRP (*p* < 0.01), fibrinogen (*p* < 0.001), and ESR (*p* < 0.05). Similar results were found between LDL score and inflammatory markers. However, the large LDL cholesterol, which has been supposed to be less atherogenic than small LDL cholesterol, was not significantly linked with any inflammatory markers in the current study (*p* > 0.05, all). We further assessed the correlation between inflammatory markers and mean LDL particle size. Interestingly, our data indicated a definitely negative association (WBC count: *p* < 0.01; lymphocyte count: *p* < 0.01; hs-CRP: *p* < 0.05; fibrinogen: *p* < 0.01; and ESR: *p* < 0.05).

Additionally, in an analysis covering HDL subfractions, multiple inflammatory markers were correlated inversely with large HDL cholesterol (WBC count: *p* < 0.01; lymphocyte count: *p* < 0.05; hs-CRP: *p* < 0.05; fibrinogen: *p* < 0.05; and D-dimer: *p* < 0.05) but not with intermediate HDL cholesterol (only hs-CRP: *p* < 0.05) and small HDL cholesterol (*p* > 0.05, all).

### 3.3. Relation of Multimarker Inflammatory Index to Lipoprotein Subfractions

Of the individual inflammatory markers, WBC count, hs-CRP, fibrinogen, and ESR were closely related to atherogenic lipoprotein subfractions; therefore, we extracted a multimarker inflammatory index weighting the coefficients of the four individual markers. Consequently, we divided this multimarker index into quartiles. As indicated in Table 3, in a model adjusting for age, sex, body mass index, hypertension, diabetes, smoking, and incidence of CAD, the levels of intermediate LDL cholesterol, small LDL cholesterol, and LDL score were dramatically increased while the mean LDL particle size was decreased according to multimarker index quartiles (*p* < 0.01, all). Besides that, the large HDL cholesterol levels were markedly declined by multimarker index quartiles (*p* < 0.05).

### 3.4. Subgroup Analysis in Patients with or without CAD

**Figure 2:**
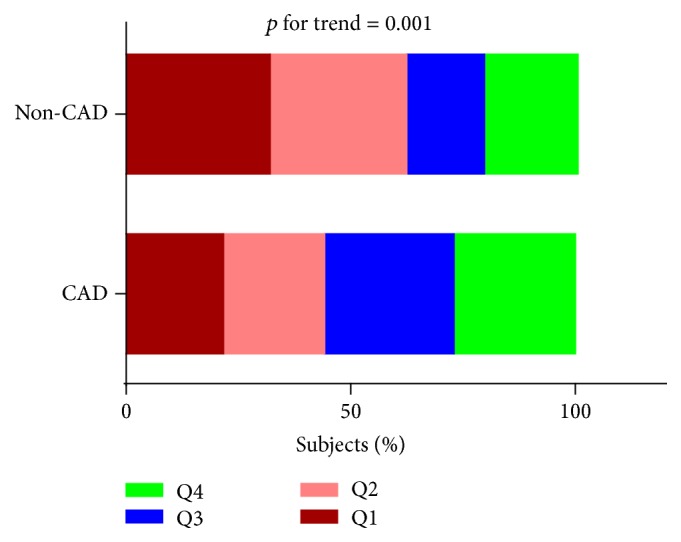
The distribution of subjects in CAD and non-CAD group across multimarker inflammatory index quartiles. Chi-squared test was performed.

**Figure 3:**
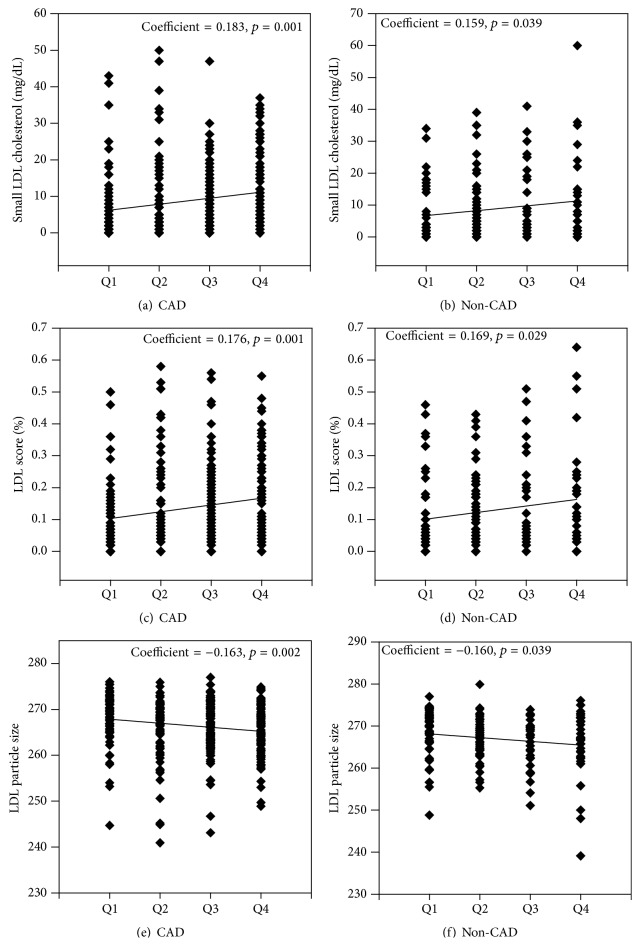
Relation of small LDL-C, LDL score, and mean LDL particle size to the quartiles of multimarker inflammatory index in patients with or without CAD ((a, b) small LDL-C; (c, d) LDL score; (e, f) mean LDL particle size). Simple linear regression analysis was applied.

Given that larger percentage of patients with CAD tended to have more severe inflammation (Figure 2), we further performed the subgroup analysis to explore the association between inflammation and atherogenic lipoprotein subfractions in patients with or without CAD. As indicated in Figure 3, we found positive associations of multimarker index quartiles with small LDL cholesterol (CAD: *β* = 0.183, *p* = 0.001; non-CAD: *β* = 0.159, *p* = 0.039) and LDL score (CAD: *β* = 0.176, *p* = 0.001; non-CAD: *β* = 0.169, *p* = 0.029) and negative associations with mean LDL particle size (CAD: *β* = −0.163, *p* = 0.002; non-CAD: *β* = −0.160, *p* = 0.039). Although the relationships were both significant in patients with or without CAD, the former tended to be slightly stronger in the present study.

## 4. Discussion

The present study confirms the low-grade systemic inflammatory markers are related to features of the circulating cholesterol levels. More importantly, the main and novel findings are that (1) multiple systemic inflammatory markers are positively correlated with the most atherogenic lipoprotein subfractions, such as small LDL cholesterol and LDL score; (2) they are negatively linked with mean LDL particle size; (3) and they are inversely related to the antiatherogenic subfraction, large HDL cholesterol. These findings suggest that the mutual interplay may be a potential major contributor in the development of atherosclerotic disease.

Although the notion that elevated inflammatory markers increase the risk of cardiovascular disease (CVD) has been increasingly recognized [22, 23], underlying mechanisms and pathways remain to be elucidated. Specifically, inflammation and dyslipidemia are well established cardiovascular risk factors and closely associated with each other. However, it remains unclear with regard to which comes first in the atherosclerotic process. As reported, inflammation could affect lipoprotein metabolism [12], which is reflected by decreased plasma HDL-C levels and impaired atheroprotective HDL functions [24]. Additionally, it also has been suggested that the relationship between inflammatory markers and atherosclerosis is independent of plasma lipoprotein levels [25]. Conversely, the presence of dyslipidemia itself may in turn further stimulate the inflammatory process [26]. There are evidences that inflammation could be elicited by modified lipoproteins such as oxidized LDL [27], as well as the TG-rich lipoprotein remnants [28]. Thus, the interplay between inflammation and lipid metabolism at multiple levels may exacerbate the development of atherosclerosis, resulting in a vicious cycle. However, the traditionally measured cholesterol levels in LDL and HDL particles could not capture all the high LDL or low HDL related risks [29, 30] and other alternative measures reflecting the particle have emerged in multiple studies.

Recently, lipoprotein subfractions have been suggested as a new cardiovascular risk strategy [31]. Experimental and turnover studies have raised the possibility that small LDL may be more atherogenic than buoyant LDL [7]. The Atherosclerosis Risk in Communities study including 11419 participants revealed that small dense LDL cholesterol was associated with the incident CAD [32]. Moreover, Nishikura et al. conducted a study including 190 consecutive CAD patients. During a seven-year follow-up period, small dense LDL cholesterol has been supposed to be a very promising biomarker in predicting future cardiovascular events [33]. Despite the cholesterol levels in LDL subfractions, mean LDL particle size has also been indicated to be closely related to cardiovascular mortality [34]. Currently, the link of HDL subclasses to prognosis remains controversial. Most studies including our data tended to support the idea that decreased large HDL-C level may be more atherogenic than other HDL subfractions [35, 36].

In view of these heterogeneous and complex relationships, it is important to characterize the role of individual markers of inflammation in relation to different lipoprotein subfractions. In the present study, we employed five kinds of inflammatory markers reflecting the chronic systemic inflammatory activity and found that most of the inflammatory markers are positively associated with atherogenic lipoprotein subfractions in patients that underwent coronary angiography. Of additional importance, we derived a composite marker of inflammation from a principle component analysis. In doing so, we were able to combine each of the individual markers into a single component, thereby reflecting a general marker of inflammation, permitting us to retain most of the information attributed to each marker. As a result, we observed that the atherogenic lipoprotein subfractions were significantly increased by multimarker index in patients with or without CAD, although it was slightly stronger in the CAD group. These novel findings may suggest that the correlation between inflammatory markers and atherogenic lipoprotein subfractions was stable and persistent and mildly modified by CAD status. Nonetheless, future investigations will have to verify these relationships in different populations and address the exact mechanisms.

The present study is not without limitations. First, it included patients scheduled for angiography. This hospital-based population may not be representative of a random population sample. Second, it was a cross-sectional study, so it was difficult to identify the causal or temporal relationship. Finally, we estimated the lipoprotein subfractions with Lipoprint System and the use of other methods should be investigated in the future.

## 5. Conclusion

In summary, systemic inflammatory markers are positively correlated with small LDL cholesterol and LDL score while being negatively linked with mean LDL particle size and large HDL cholesterol, highlighting the potential contribution to increased cardiovascular risk.

## Figures and Tables

**Table 1:** Baseline characteristics.

Characteristics	All subjects (*n* = 520)	CAD (*n* = 351)	Non-CAD (*n* = 169)	*p* value
*Coronary risk factors *				
Age (years)	56.6 ± 10.0	57.9 ± 9.7	54.0 ± 10.2	<0.001
Male, % (*n*)	64.4 (335)	71.8 (252)	28.2 (99)	<0.001
BMI (kg/m^2^)	25.7 ± 3.5	25.9 ± 3.4	25.4 ± 3.5	0.147
Smoking, % (*n*)	43.5 (226)	50.1 (176)	29.6 (50)	<0.001
Hypertension, % (*n*)	60.0 (312)	67.0 (235)	45.6 (77)	<0.001
Diabetes mellitus, % (*n*)	21.7 (113)	25.1 (88)	14.8 (25)	0.009
Dyslipidemia, % (*n*)	67.7 (352)	70.7 (248)	61.5 (104)	0.045
Family history of CAD, % (*n*)	16.0 (83)	17.9 (63)	11.8 (20)	0.096
*Lipoprotein parameters*				
VLDL cholesterol (mg/dL)	44.0 ± 12.3	44.0 ± 10.8	43.9 ± 14.9	0.253
IDL cholesterol (mg/dL)	48.7 ± 13.8	48.3 ± 13.4	49.4 ± 14.6	0.606
LDL cholesterol (mg/dL)	126.1 ± 38.7	125.7 ± 38.4	127.0 ± 39.3	0.723
Large LDL cholesterol	28.7 ± 10.1	28.3 ± 10.1	29.4 ± 10.1	0.277
Intermediate LDL cholesterol	20.5 ± 9.5	20.7 ± 9.5	20.1 ± 9.4	0.466
Small LDL cholesterol	8.6 ± 9.6	9.0 ± 9.8	7.9 ± 9.3	0.092
LDL score (%)	0.13 ± 0.13	0.14 ± 0.13	0.12 ± 0.13	0.067
Mean LDL particle size (Å)	266.7 ± 6.0	266.4 ± 5.9	267.2 ± 6.0	0.079
HDL cholesterol (mg/dL)	43.3 ± 13.9	42.5 ± 14.2	45.1 ± 13.1	0.054
Large HDL cholesterol	14.0 ± 7.4	13.5 ± 7.2	15.1 ± 7.7	0.027
Intermediate HDL cholesterol	20.9 ± 6.5	20.7 ± 7.0	21.2 ± 5.3	0.360
Small HDL cholesterol	8.6 ± 3.3	8.5 ± 3.4	8.8 ± 3.1	0.318
*Inflammatory markers*				
WBC count (×10^9^/L)	6.1 ± 1.7	6.2 ± 1.8	5.9 ± 1.4	0.076
Neutrophil count (×10^9^/L)	3.7 ± 1.4	3.8 ± 1.4	3.5 ± 1.1	0.019
Lymphocyte count (×10^9^/L)	1.9 ± 0.6	1.9 ± 0.6	2.0 ± 0.5	0.260
Monocyte count (×10^9^/L)	0.39 ± 0.29	0.40 ± 0.34	0.37 ± 0.12	0.147
hs-CRP (mg/L)	2.6 ± 9.8	2.8 ± 3.1	2.2 ± 2.8	<0.001
Fibrinogen (g/L)	3.1 ± 0.8	3.2 ± 0.8	3.0 ± 0.6	0.001
ESR (mm/h)	9.8 ± 9.7	10.1 ± 10.2	9.1 ± 8.6	0.463
D-dimer (*μ*g/mL)	0.37 ± 0.44	0.40 ± 0.37	0.33 ± 0.56	0.115

Data are expressed as % (*n*), median (IQR), or mean ± SD. BMI: body mass index; CAD: coronary artery disease; VLDL: very low-density lipoprotein; IDL: intermediate-density lipoprotein; LDL: low-density lipoprotein; HDL: high-density lipoprotein; WBC: white blood cell; hs-CRP: high sensitivity C-reactive protein; ESR: erythrocyte sedimentation rate.

**Table 2:** Age- and sex-adjusted correlations between lipoprotein subfractions and inflammatory markers.

	WBC count	Neutrophil count	Lymphocyte count	Monocyte count	hs-CRP	Fibrinogen	ESR	D-dimer
VLDL cholesterol (mg/dL)	0.133^*∗∗*^	0.112^*∗*^	0.124^*∗∗*^	−0.019	0.129^*∗∗*^	0.189^*∗∗∗*^	0.154^*∗∗∗*^	−0.055
IDL cholesterol (mg/dL)	0.055	0.080	0.009	−0.053	0.115^*∗∗*^	0.129^*∗∗*^	0.090^*∗*^	−0.042
LDL cholesterol (mg/dL)	0.078	0.079	0.053	−0.028	0.112^*∗*^	0.090^*∗*^	0.020	−0.056
Large LDL cholesterol	−0.054	−0.020	−0.075	−0.059	0.008	−0.031	−0.064	−0.008
Intermediate LDL cholesterol	0.094^*∗*^	0.072	0.102^*∗*^	0.001	0.153^*∗∗∗*^	0.095^*∗*^	0.045	−0.077
Small LDL cholesterol	0.128^*∗∗*^	0.099^*∗*^	0.120^*∗∗*^	0.007	0.121^*∗∗*^	0.141^*∗∗∗*^	0.111^*∗*^	−0.054
LDL score (%)	0.124^*∗∗*^	0.095^*∗*^	0.111^*∗*^	0.009	0.099^*∗*^	0.139^*∗∗*^	0.125^*∗∗*^	−0.057
Mean LDL particle size (Å)	−0.115^*∗∗*^	−0.084	−0.117^*∗∗*^	−0.016	−0.096^*∗*^	−0.117^*∗∗*^	−0.111^*∗*^	0.071
HDL cholesterol (mg/dL)	−0.093^*∗*^	−0.064	−0.072	−0.059	−0.106^*∗*^	−0.075	−0.034	0.063
Large HDL cholesterol	−0.123^*∗∗*^	−0.089^*∗*^	−0.104^*∗*^	−0.052	−0.085	−0.092^*∗*^	−0.060	0.104^*∗*^
Intermediate HDL cholesterol	−0.077	−0.055	−0.053	−0.053	−0.090^*∗*^	−0.047	−0.029	0.042
Small HDL cholesterol	−0.013	−0.006	−0.001	−0.036	−0.085	−0.057	0.004	−0.061

Partial correlations are shown. All the correlations were adjusted for age and sex. VLDL: very low-density lipoprotein; IDL: intermediate-density lipoprotein; LDL: low-density lipoprotein; HDL: high-density lipoprotein; WBC: white blood cell; hs-CRP: high sensitivity C-reactive protein; ESR: erythrocyte sedimentation rate.

^*∗*^ *p* < 0.05.

^*∗∗*^ *p* < 0.01.

^*∗∗∗*^ *p* < 0.001.

**Table 3:** Relationship of LDL and HDL subfractions with multimarker inflammatory index.

	Multimarker inflammatory index				
	Q1 (*n* = 130)	Q2 (*n* = 130)	Q3 (*n* = 130)	Q4 (*n* = 130)	*p* for trend
*LDL subfractions*					
Large LDL cholesterol (mg/dL)					
Model 1	30.07 ± 0.89	27.92 ± 0.89	28.44 ± 0.89	28.03 ± 0.89	0.289
Model 2	30.36 ± 0.89	27.84 ± 0.89	28.30 ± 0.89	27.96 ± 0.89	0.157
Model 3	30.01 ± 0.91	27.89 ± 0.89	28.53 ± 0.91	28.06 ± 0.90	0.338
Intermediate LDL cholesterol (mg/dL)					
Model 1	18.02 ± 0.83	20.76 ± 0.83	21.19 ± 0.83	22.02 ± 0.83	0.005
Model 2	18.03 ± 0.84	20.62 ± 0.83	21.29 ± 0.84	22.06 ± 0.83	0.005
Model 3	18.06 ± 0.85	20.72 ± 0.83	21.23 ± 0.85	22.01 ± 0.83	0.009
Small LDL cholesterol (mg/dL)					
Model 1	5.87 ± 0.87	8.92 ± 0.87	9.46 ± 0.87	10.89 ± 0.87	0.001
Model 2	5.73 ± 0.87	8.72 ± 0.87	9.69 ± 0.87	11.00 ± 0.87	<0.001
Model 3	5.79 ± 0.90	8.74 ± 0.87	9.64 ± 0.89	10.98 ± 0.87	0.001
LDL score (%)					
Model 1	0.09 ± 0.01	0.14 ± 0.01	0.15 ± 0.01	0.16 ± 0.01	<0.001
Model 2	0.09 ± 0.01	0.13 ± 0.01	0.15 ± 0.01	0.16 ± 0.01	<0.001
Model 3	0.10 ± 0.01	0.13 ± 0.01	0.15 ± 0.01	0.16 ± 0.01	0.001
Mean LDL particle size (Å)					
Model 1	268.42 ± 0.52	266.38 ± 0.52	266.21 ± 0.52	265.51 ± 0.52	0.001
Model 2	268.54 ± 0.53	266.44 ± 0.52	266.09 ± 0.53	265.45 ± 0.52	<0.001
Model 3	268.39 ± 0.54	266.45 ± 0.52	266.18 ± 0.53	265.49 ± 0.52	0.001

*HDL subfractions*					
Large HDL cholesterol (mg/dL)					
Model 1	15.29 ± 0.64	13.57 ± 0.64	14.03 ± 0.64	13.02 ± 0.64	0.080
Model 2	15.97 ± 0.61	13.75 ± 0.61	13.44 ± 0.61	12.73 ± 0.61	0.002
Model 3	15.33 ± 0.60	13.62 ± 0.59	13.95 ± 0.60	13.04 ± 0.59	0.049
Intermediate HDL cholesterol (mg/dL)					
Model 1	21.69 ± 0.57	20.70 ± 0.57	20.27 ± 0.57	20.65 ± 0.57	0.329
Model 2	22.07 ± 0.56	20.71 ± 0.56	20.02 ± 0.56	20.52 ± 0.56	0.066
Model 3	21.96 ± 0.57	20.68 ± 0.56	20.08 ± 0.57	20.58 ± 0.56	0.129
Small HDL cholesterol (mg/dL)					
Model 1	8.52 ± 0.29	8.84 ± 0.29	8.49 ± 0.29	8.40 ± 0.30	0.740
Model 2	8.56 ± 0.30	8.79 ± 0.29	8.50 ± 0.30	8.40 ± 0.29	0.815
Model 3	8.63 ± 0.30	8.80 ± 0.29	8.42 ± 0.30	8.37 ± 0.29	0.729

Values were obtained from general linear models. Model 1 was unadjusted. Model 2 was adjusted for age and sex. Model 3 was additionally adjusted for BMI, hypertension, diabetes, smoking, and incidence of CAD. BMI: body mass index; CAD: coronary artery disease; LDL: low-density lipoprotein; HDL: high-density lipoprotein.
